# Competitive Exclusion Is a Major Bioprotective Mechanism of Lactobacilli against Fungal Spoilage in Fermented Milk Products

**DOI:** 10.1128/AEM.02312-19

**Published:** 2020-03-18

**Authors:** Solvej Siedler, Martin Holm Rau, Susanne Bidstrup, Justin M. Vento, Stina Dissing Aunsbjerg, Elleke F. Bosma, Laura M. McNair, Chase L. Beisel, Ana Rute Neves

**Affiliations:** a Discovery, R&D, Chr. Hansen A/S, Hørsholm, Denmark; b Department of Chemical and Biomolecular Engineering, North Carolina State University, Raleigh, North Carolina, USA; c Global Application, Chr. Hansen A/S, Hørsholm, Denmark; d Department of Drug Design and Pharmacology, University of Copenhagen, Copenhagen, Denmark; e Helmholtz Institute for RNA-based Infection Research (HIRI), Helmholtz Center for Infection Research, Würzburg, Germany; f Medical Faculty, University of Würzburg, Würzburg, Germany; University of Bayreuth

**Keywords:** lactic acid bacteria, bioprotection, food spoilage, manganese starvation, genome editing, *Lactobacillus*

## Abstract

A prominent feature of lactic acid bacteria (LAB) is their ability to inhibit growth of spoilage organisms in food, but hitherto research efforts to establish the mechanisms underlying bioactivity focused on the production of antimicrobial compounds by LAB. We show, in this study, that competitive exclusion, i.e., competition for a limited resource by different organisms, is a major mechanism of fungal growth inhibition by lactobacilli in fermented dairy products. The depletion of the essential trace element manganese by two *Lactobacillus* species was uncovered as the main mechanism for growth inhibition of dairy spoilage yeast and molds. A manganese transporter (MntH1), representing one of the highest expressed gene products in both lactobacilli, facilitates the exhaustive manganese scavenging. Expression of the *mntH1* gene was found to be strain dependent, affected by species coculturing and the growth phase. Further, deletion of the *mntH1* gene in one of the strains resulted in a loss of bioactivity, proving this gene to be important for manganese depletion. The presence of an *mntH* gene displayed a distinct phylogenetic pattern within the *Lactobacillus* genus. Moreover, assaying the bioprotective ability in fermented milk of selected lactobacilli from 10 major phylogenetic groups identified a correlation between the presence of *mntH* and bioprotective activity. Thus, manganese scavenging emerges as a common trait within the *Lactobacillus* genus, but differences in expression result in some strains showing more bioprotective effect than others. In summary, competitive exclusion through ion depletion is herein reported as a novel mechanism in LAB to delay the growth of spoilage contaminants in dairy products.

**IMPORTANCE** In societies that have food choices, conscious consumers demand natural solutions to keep their food healthy and fresh during storage, simultaneously reducing food waste. The use of “good bacteria” to protect food against spoilage organisms has a long, successful history, even though the molecular mechanisms are not fully understood. In this study, we show that the depletion of free manganese is a major bioprotective mechanism of lactobacilli in dairy products. High manganese uptake and intracellular storage provide a link to the distinct, nonenzymatic, manganese-catalyzed oxidative stress defense mechanism, previously described for certain lactobacilli. The evaluation of representative *Lactobacillus* species in our study identifies multiple relevant species groups for fungal growth inhibition via manganese depletion. Hence, through the natural mechanism of nutrient depletion, the use of dedicated bioprotective lactobacilli constitutes an attractive alternative to artificial preservation.

## Abstract (precis)

In societies that have food choices, conscious consumers demand natural solutions to keep their food healthy and fresh during storage, simultaneously reducing food waste. The use of “good bacteria” to protect food against spoilage organisms has a long, successful history, even though the molecular mechanisms are not fully understood. In this study, we show that the depletion of free manganese is a major bioprotective mechanism of lactobacilli in dairy products. High manganese uptake and intracellular storage provide a link to the distinct, nonenzymatic, manganese-catalyzed oxidative stress defense mechanism, previously described for certain lactobacilli. The evaluation of representative *Lactobacillus* species in our study identifies multiple relevant species groups for fungal growth inhibition via manganese depletion. Hence, through the natural mechanism of nutrient depletion, the use of dedicated bioprotective lactobacilli constitutes an attractive alternative to artificial preservation.

## INTRODUCTION

Growing demand for healthy and fresh foods without added artificial preservatives is increasing the need for natural, microbial solutions. Among microbes, lactic acid bacteria (LAB) have a long history in protecting food from spoilage, and, today, bioprotective bacteria that specifically inhibit the growth of spoilage organisms have emerged as an alternative way of keeping food fresh for longer periods of time (1).

More than 200 species are covered by the exceptionally diverse genus *Lactobacillus*, with other genera such as *Pediococcus* phylogenetically intermixed (2). Believed to originally be free living, lactobacilli today also include numerous host-adapted species, with habitats in livestock feed, plants, animals, humans, and fermented foods (3). Several species have a generally recognized as safe (GRAS) status and are applied within the dairy industry for the production of fermented milk products, and certain members are widely applied as probiotics; among these are especially Lactobacillus rhamnosus and Lactobacillus paracasei (4, 5). These two species display a more nomadic lifestyle capable of colonizing the human and animal gastrointestinal tract while also displaying efficient growth in milk.

In dairy products, spoilage by mold and yeast cells is one of the major problems that reduces shelf life (6). As a nutrient-rich environment, yogurt is inherently susceptible to microbial spoilage, although certain characteristics narrow the number of potential detrimental organisms. The innate carbon source is lactose; free amino acids are scarce and are instead concentrated in caseins, which require proteolysis for liberation, and the starter cultures responsible for conversion of milk to yogurt lower the pH to around 4.5. The typically observed spoilage organisms consist of various yeasts such as Debaryomyces hansenii, Saccharomyces cerevisiae, and several others, in addition to molds, especially of the *Penicillium* genus (6).

In the past decade, the bioprotective potential of LAB has spurred considerable efforts in the scientific community to identify new strains with bioprotective properties from various food sources (7–9), as well as attempts to elucidate the mechanisms behind the observed bioactivity (10–12). Numerous metabolites produced by LAB have been identified as having antifungal and antibacterial activities (13), although *in situ* concentrations are typically significantly below MICs (14). Meanwhile, other mechanisms have, until now, been largely unexplored.

Competitive exclusion is a widespread phenomenon in nature and includes the competition for nutrients such as a carbon source (15, 16); for a physical space, such as in the gastrointestinal tract (17); as well as for essential ions. The concept of nutritional immunity in the human body (18) is an example of competition for essential ions, reinforced by the various intricate means of iron scavenging through the action of specific siderophores present in bacterial pathogens (19).

Manganese (Mn) is an essential trace element that is a key cofactor in all kingdoms of life, making it important for the growth of bacteria, yeast, and mold (20). The two major manganese uptake systems in LAB are the NRAMP-type transporter MntH and the ABC transporter SitABC. While the ABC transporter is mainly active at neutral pH, the proton-driven symporter MntH is the major transport system under acidic conditions (21).

In Escherichia coli, manganese is a critical cofactor for the superoxide dismutase (SOD) protein (22). Aerotolerant lactic acid bacteria generally lack an active SOD gene and cope with reactive oxygen species through an alternative mechanism, which has mainly been studied in Lactobacillus plantarum (23). In this bacterium, intracellular manganese(II), accumulated in excessively high concentrations of up to 25 mM manganese, scavenges oxygen radicals (O_2_^−^) as effectively as SOD (23). This defense mechanism has been shown for other lactobacilli species (e.g., Lactobacillus casei and Lactobacillus fermentum) but is not present in all members of the genus (e.g., Lactobacillus bulgaricus and Lactobacillus acidophilus) (23). The manganese(II) in L. plantarum primarily associates with a large complex of nondialyzable polyphosphate-protein aggregates (24). The nonenzymatic manganese protection against superoxide *in vivo* was described in the early 1980s (23), but the mechanism was shown only recently *in vitro* (25). So far, reports of the mechanism in other bacteria are limited, although, in some organisms, it could serve as auxiliary protection (26). High intracellular manganese(II) concentration has, for example, previously been associated with other stress defense mechanisms, such as radiation resistance (27).

In this study, we show that the competition for manganese is a major limiting factor for the growth of spoilage organisms in yogurt containing a bioprotective culture of L. paracasei and L. rhamnosus. Furthermore, we investigated the bioprotective potential of the *Lactobacillus* genus based on this novel competition mechanism.

## RESULTS

### Manganese is the limiting factor for yeast growth in yogurt containing a bioprotective culture.

To analyze the activity of the bioprotective culture in yogurt in an easy and high-throughput manner, we developed an assay to determine the bioactivity based on yeast growth measured by absorbance at 600 nm. For this, we centrifuged the yogurt and filtered the supernatant to obtain a clear aqueous phase (AQ) of the yogurt. The AQ was inoculated with yeast cells, and the yeast growth was detected by absorbance measurement in a microplate reader after several days of incubation at 17°C. Analysis of D. hansenii growth by enumeration of cells was comparable in yogurt and AQ. The AQ of the reference (REF AQ) reached slightly lower cell counts, possibly due to a smaller amount of proteins in the AQ (Fig. S1). The yeast D. hansenii was chosen as the reference organism, as it is a common spoilage organism and is sensitive to bioprotective cultures in yogurt (12).

**FIG 1:**
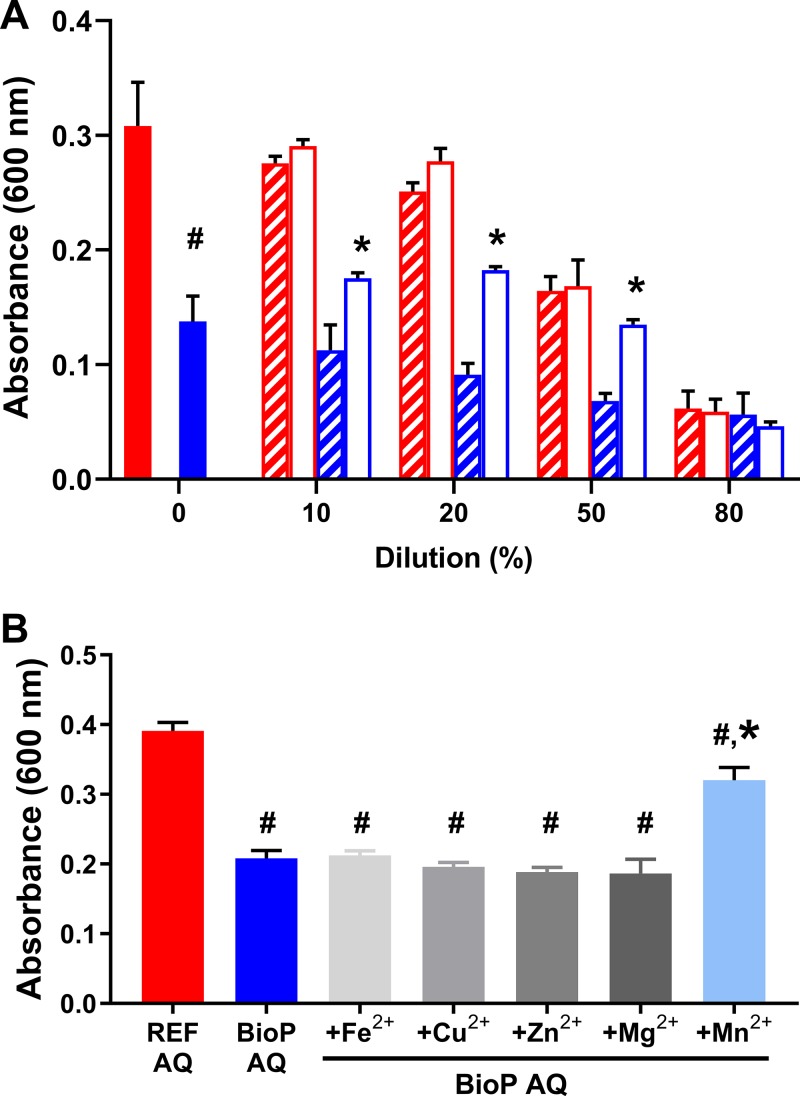
Growth of D. hansenii in aqueous phase (AQ) of reference (REF) yogurt (red bars) and bioprotective strains (BioP) containing yogurt (blue bars). (A) The REF AQ and BioP AQ were diluted with tap water (clear bars) or MilliQ water (striped bars). Growth of D. hansenii was measured at 600 nm after 4 days of incubation at 17°C. (B) Effect of complementation BioP AQ with metals found in milk on the growth of D. hansenii (light blue bars). Growth was measured after 5 days of incubation at 17°C and compared to REF AQ without any addition of metal ions. Metal concentrations were used as found in milk (0.3 mg/liter Fe^2+^, 0.1 mg/liter Cu^2+^, 4.2 mg/liter Zn^2+^, 60 mg/liter Mg^2+^, and 0.03 mg/liter Mn^2+^). Mean and standard deviation of three replicates are indicated by the bars and error bars. Hashtags (#) indicate data being statistically significantly different (*P* < 0.0001) from REF AQ (A, Student's *t* test; B, one-way ANOVA), and asterisks (*) indicate data being different from BioP AQ diluted with MilliQ water (A, two-way ANOVA) or undiluted/nonsupplemented BioP AQ (B, one-way ANOVA).

Here, the yogurt bioprotective culture consists of an L. rhamnosus strain and an L. paracasei strain, collectively designated BioP. A significant difference between D. hansenii growth in AQ of yogurt with (BioP AQ) and without (REF AQ) bioprotective culture was detected (*P* < 0.0001; *n* = 6, *t* = 9.292, df = 10; Student's *t* test, two tailed) (Fig. 1). Moreover, we noticed a difference in the bioactivity upon 10 to 50% (not 80%) dilution of BioP AQ with either tap water or MilliQ water (*P* < 0.0001; *n* = 3), while similar growth reduction was seen when diluting the REF AQ, i.e., there was no significant difference between D. hansenii growth levels in REF AQ when diluted 10 to 80% with tap water or MilliQ water (*P* > 0.05, *n* = 3; df = 32; two-way analysis of variance [ANOVA]; Fig. 1A). The decreased yeast growth in MilliQ-diluted samples suggests a dilution of a bioactive compound, but the results using tap water point to a lack of minerals in the BioP sample that can be replenished by tap water. To test which factor could be limiting, we added different metals to BioP AQ which could be present in tap water but filtered out in MilliQ water. In our assay, the added metal ion concentrations were in the same range as those present in milk (28, 29). Of all tested metals, only the addition of manganese resulted in the growth of D. hansenii that was comparable to growth in the REF AQ (Fig. 1B). In particular, the growth in BioP AQ (*n* = 5) and BioP AQ supplemented with iron, copper, zinc, or magnesium ions (*n* = 2 to 5) was not significantly different (*P* > 0.05), whereas the growth in BioP AQ and BioP AQ supplemented with manganese ions (*n* = 4) was significantly different (*P* < 0.0001; df = 19; one-way ANOVA). This indicates the availability of manganese ions as the sole limiting factor in the BioP AQ. We investigated the minimal concentration needed to promote growth of two different D. hansenii strains in BioP AQ (Fig. S2) and in a chemically defined medium (Fig. S3), and we identified a threshold of ∼0.01 mg/liter under both conditions. Furthermore, we measured the manganese concentration in the AQs, which was 0.03 mg/liter in REF AQ and below the quantification limit of 0.003 mg/liter in BioP AQ.

### Inhibition of different yeast and mold strains.

**FIG 2:**
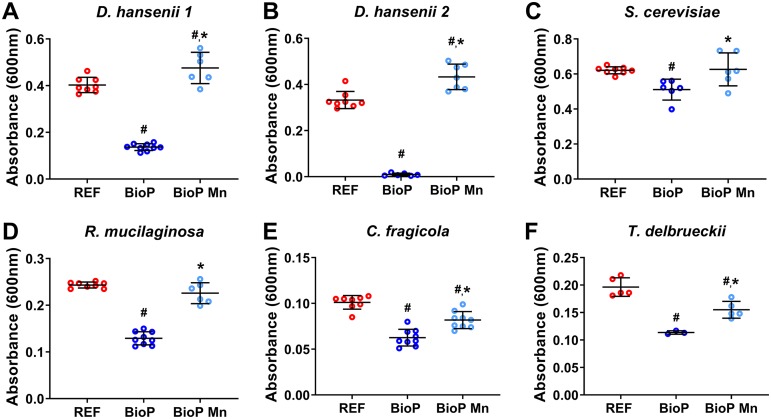
Yeast growth in BioP AQ without (dark blue) and with (light blue) the addition of 0.6 mg/liter manganese and in REF AQ (red) after 5 days of incubation at 17°C. The following yeasts were tested: D. hansenii strain 1 (A), D. hansenii strain 2 (B), S. cerevisiae (C), R. mucilaginosa (D), C. fragicola (E), and T. delbrueckii (F). Individual data points for the 6 to 9 replicates are shown along with indications of mean ± standard deviation. Hashtags (#) indicate data being statistically significantly different (*P* < 0.05) from REF AQ, and asterisks (*) indicate data being different from nonsupplemented BioP AQ (one-way ANOVA).

After investigating the effect of manganese on the growth of D. hansenii in BioP AQ, we analyzed the impact of manganese on different yeast strains. All yeast strains comprise food isolates that are relevant for food spoilage. The growth of all tested yeast strains was inhibited in the BioP AQ compared to REF AQ (#, *P* < 0.0001; *n* = 3 to 9; df = 10 to 23; one-way ANOVA in Fig. 2). The addition of manganese ions fully restored the growth of S. cerevisiae and Rhodotorula mucilaginosa in BioP AQ. In particular, no significant difference was detected between REF AQ and BioP AQ supplemented with manganese ions regarding the growth of R. mucilaginosa (*P* = 0.0990) and S. cerevisiae (*P* = 0.9860). In contrast, the growth of Cryptococcus fragicola, Torulaspora delbrueckii, and the two D. hansenii strains was significantly different in BioP AQ supplemented with manganese ions in comparison with REF AQ (#, *P* < 0.05 in Fig. 2). Nevertheless, significant differences were also observed between growth levels of these spoilage yeasts in BioP AQ with and without added manganese ions added (*, *P* < 0.05 in Fig. 2), supporting the view that the lack of free manganese ions in BioP AQ underlies the majority of its bioactivity. D. hansenii 1 growth was not significantly different between REF AQ and REF AQ with manganese addition (*P* = 0.1112) or REF AQ and BioP AQ both supplemented with manganese (*P* = 0.4442) (Fig. S4). We also tested the D. hansenii type strain (ATCC 36239), but the experiments with this strain were hampered due to its inability to grow in REF AQ. This exemplifies the need for relevant food isolates, as many of the strains intended for academic use have lost phenotypic traits that are key for industrial applications.

**FIG 3:**
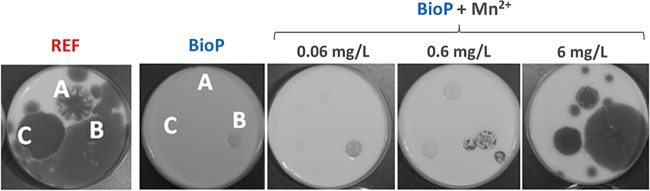
Growth of 3 different molds: P. brevicompactum (A), P. crustosum (B), and P. solitum (C) on plates prepared from milk fermented with starter culture alone (REF) or both starter and bioprotective culture (BioP). Different manganese concentrations were added as indicated. The spoilage molds were added in concentrations of 500 spores/spot. The plates were incubated at 22°C for 8 days.

After showing the effect of limited manganese availability on yeast growth, we tested the effect of manganese on the growth of three different spoilage molds from the family *Penicillium*. These strains display various levels of sensitivity to bioprotective cultures in yogurt. Mold spores were spotted on the top of agar-solidified yogurt with and without bioprotective culture, and the plates were incubated for 8 days at room temperature. The sample with the bioprotective culture showed clear inhibition of the mold growth compared to the reference. The addition of increasing manganese concentrations of up to 6 mg/liter correlated positively with mold growth (Fig. 3).

### Identification of bioprotective-relevant genes by transcriptomics.

**FIG 4:**
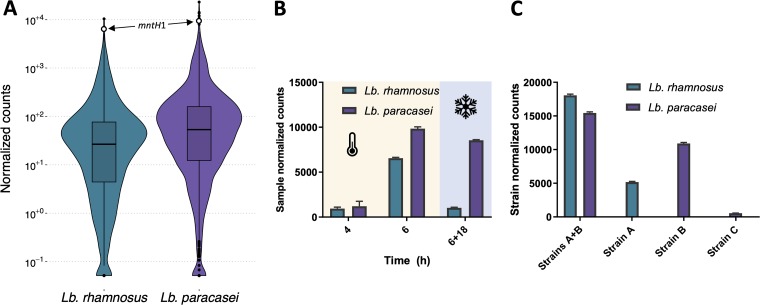
Overall and *mntH1*-specific gene count distribution. (A) Sample-normalized gene count distribution of BioP L. rhamnosus and L. paracasei during coculture after 6 h milk fermentation. A white circle denotes the *mntH1* count level. (B) Temporal, sample-normalized *mntH1* count levels of BioP strains during 37°C milk fermentation (4 h, 6 h), and after 6 h of 37°C milk fermentation and 18 h of 7°C storage (6 + 18 h). (C) Normalized *mntH1* count levels after 6 h of coculture (strains A and B) and individual culture (strain A, strain B, and strain C) in milk. Normalization is here performed individually for each strain, also for coculture. Included are the BioP strains (strains A and B) and an L. paracasei strain with a lower level of bioprotective activity (strain C).

As different mechanisms of the observed manganese depletion could be hypothesized, for instance, through sequestration or uptake, we performed global gene expression profiles of the two *Lactobacillus* strains with bioprotective properties to examine the underlying mechanism. Experiments were performed in milk, but the starter culture required for yogurt production was omitted, as the relative abundance of BioP strain transcripts was comparatively marginal in yogurt. The depicted gene count distribution (Fig. 4A) of the cocultured BioP strains provides an example of the large count range that is typical of RNA sequencing (RNA-seq) data (30), here more than 5 orders of magnitude. For both strains, among the most expressed genes was an *mntH* manganese transporter-encoding gene (*mntH1*), remarkably displaying fifth- and seventh-highest read counts of all genes in L. rhamnosus and L. paracasei, respectively. *mntH1* expression was magnitudes above the median (>100-fold) and putatively constitutes up to 1.8% of all transcripts. Such high expression, as judged from gene counts, of a manganese transporter gene is atypical. Other genes among the top 10 expressed relate to well-established highly expressed functions such as glycolysis or translation. Apart from the specified *mntH1* gene, the genomes of L. rhamnosus and L. paracasei harbor one and two additional *mntH* homologs (*mntH*2 and *mntH*3), respectively. The two specified *mntH1* genes are orthologs and are, in both species, the most distal *mntH* gene to the origin of replication in the clockwise direction. For the additional *mntH* paralogs, the expression is more than 200-fold lower; hence, their products are unlikely to contribute significantly to manganese uptake. Additionally, both genomes harbor the ABC manganese transport system (*sitABC*) with 20- and 10-fold lower-expression than the key *mntH1* gene, respectively, in L. rhamnosus and L. paracasei (Table S1).

The temporal *mntH1* expression (Fig. 4B) revealed a marked increase from 4 to 6 h of milk fermentation (false-discovery rate [FDR] < 10^−37^) and with the expression remaining high during cold storage (400 to 500 times above median transcript levels), which indicates continued manganese uptake capacity, even during product shelf life. This correlates with an increase in bioactivity during storage (Fig. S5). Apart from temporal expression changes, *mntH1* expression also seems to be a function of strain genotype and coculturing (Fig. 4C). Culturing strains individually led to lower *mntH1* expression for both *Lactobacillus* strains than coculturing. Apart from coculturing, the effect of genotype on *mntH1* expression seems to be correlated with bioprotective activity. L. paracasei strain C, for instance, was included based on its lower bioprotective activity (data not shown) and, in fact, displays a markedly lower *mntH1* expression level (20-fold) than strain B, the corresponding BioP L. paracasei (Fig. 4C). In yogurt, two starter culture species (Streptococcus thermophilus and Lactobacillus delbrueckii) are ordinarily present. Of the two, only S. thermophilus also harbors an *mntH* gene. The relative expression of this gene in a representative strain is around 25-fold lower than the BioP strains (data not shown) and thereby comparable to L. paracasei strain C.

### MntH1 is essential for bioprotective activity.

**FIG 5:**
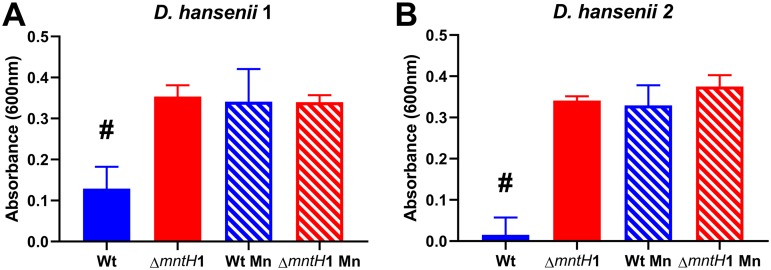
Effect of *mntH1* deletion on yeast growth inhibition. Yeast growth in AQ of milk fermented by wild-type L. paracasei strain B (WT) and Δ*mntH1* L. paracasei strain B, without (full bars) and with 0.6 mg/liter manganese addition (dashed bars). Two yeast, D. hansenii 1 (A) and D. hansenii 2 (B), were included, and the growth was measured after 5 days of incubation at 17°C. Means and standard deviations of two biological replicates are indicated by bars and error bars. Hashtags (#) indicate data being statistically significantly different (*P* < 0.0001) from WT with manganese (Student's *t* test).

As the expression data provided strong indications of the importance of MntH1 for manganese uptake and the associated bioprotective phenotype, we sought to verify this through deletion of the *mntH1* gene in the BioP L. paracasei strain B using a homologous recombination strategy coupled with CRISPR-Cas9-mediated mutant selection (Fig. S6). The bioprotective ability of the Δ*mntH1* mutant was evaluated by assessing yeast growth in the AQ of milk fermented by individual strains (Fig. 5).

While the milk fermentation profile was identical for the wild-type (WT) BioP strain B and its Δ*mntH1* mutant (Fig. S7), yeast growth was only inhibited in the AQ of milk fermented by the WT BioP strain B and not by the corresponding Δ*mntH1* mutant. The addition of 0.6 mg/liter manganese resulted in restored yeast growth for WT BioP strain B, comparable to the growth in the AQ of milk fermented by the corresponding Δ*mntH1* mutant with and without manganese addition. Complementation of the Δ*mntH1* mutant with a plasmid containing the *mntH1* gene under its own promoter fully restored bioactivity, while an empty plasmid control did not (Fig. S8). These results provide further evidence that *mntH1* is responsible for the bioprotective phenotype. Culturing strains individually for a longer time in this experiment provides the strains with an increased potential for manganese uptake, compared to the coculture with starter culture normally performed for yogurt production. Even so, yeast inhibition by manganese uptake is insufficient in the Δ*mntH* strain to inhibit the growth of D. hansenii, despite the existence of two alternative manganese transporters within the genome.

### Correlation between bioprotective activity and *mntH* phylogenetic distribution.

**FIG 6:**
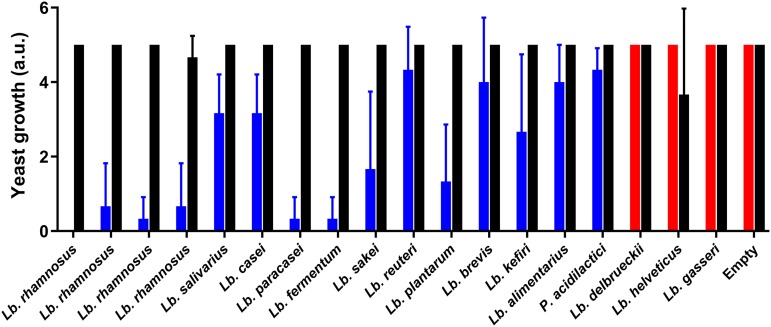
Growth scores of D. hansenii after 4 days of incubation at 17°C in fermented milk with a strain containing (blue) and not containing (red) an *mntH* gene. The addition of 6 mg/liter manganese restored the yeast growth in all cases (black). The average and standard deviation of two biological independent experiments are shown (*n* = 2).

As the MntH1 transporter seems to be heavily involved in the increased uptake of manganese in certain lactobacilli, we investigated the presence of any *mntH* homologs in different species of the genus *Lactobacillus* (Fig. S9). In total, 18 strains were selected from 15 species, covering the major phylogenetic groups of the *Lactobacillus sensu lato* species tree (3). Of these, 15 strains harbored an *mntH* homolog, while 3 strains did not. In brief, a phylogenetic basis seems to exist for the distribution of an *mntH* gene among the lactobacilli. For the phylogenetic group containing the dairy-associated species, L. delbrueckii subsp. *bulgaricus*, Lactobacillus gasseri, and Lactobacillus helveticus, a homolog is absent. In contrast, in representatives of the nine other studied groups, it is present. The correlation between *mntH* presence and yeast inhibition was subsequently investigated experimentally. We performed milk acidification with a starter culture in the presence of the different lactobacilli. The fermented milk was divided into two samples, and 6 mg/liter manganese was added to one sample, while the other sample was left unchanged. A bioactivity assay following D. hansenii growth in both conditions was performed (Fig. 6). The majority of the tested strains containing an *mntH* gene were bioactive, at various levels, while those without the gene were not bioactive. The presence of the manganese ABC transporter did not correlate better with activity, as it is only present in L. rhamnosus, L. casei, L. paracasei, and L. plantarum. The addition of manganese restored the growth of D. hansenii completely in all samples, proving this metal to be the limiting factor.

## DISCUSSION

In this study, we found that the ability of diverse lactobacilli to scavenge manganese results in inhibited growth of yeast and mold in fermented milk. Several studies have previously investigated the ability of lactobacilli to inhibit fungi in various foods, and the usage of lactobacilli for this purpose in commercial applications is gaining considerable interest. Thus far, bacterial production of inhibitory metabolites has been the focus of most studies, but the identified compounds are mostly found in concentrations markedly below the corresponding MIC of the metabolite (14). Our study shows that competitive exclusion is a major mechanism employed by many of lactobacilli to inhibit fungal growth in fermented milk. As a mechanism to avoid unwanted fungal growth, competitive exclusion is known from the concept of “nutritional immunity” in the human body, where depletion of iron and zinc in the respective parts of the body results in inhibition of pathogenic fungal and bacterial growth (18). In the context of lactic acid bacteria, examples of competitive exclusion of nutrients are scarce. A study by Honoré et al. (16) found that L. paracasei-mediated consumption of glucose and certain amino acids in a chemically defined medium correlated with reduced mold growth on the spent culture medium.

Here, the reduction of available manganese was proven as an effective inhibition mechanism against all the tested yeasts and molds. Yet two yeast strains, T. delbrueckii and C. fragicola, only displayed partially restored growth upon manganese addition (Fig. 2), indicating that other mechanisms, such as higher acid production or other antimicrobial compounds, could be involved as well (31). In milk, manganese is one of the essential trace metals with the lowest concentration (28, 29), and, consequently, the bacterial consumption required to reach a growth inhibitory concentration is low. Unlike the addition of other ions, the addition of 0.03 mg/liter manganese restored D. hansenii growth. This correlates well with the determined minimal manganese concentration threshold for D. hansenii growth of ∼0.01 mg/liter. In contrast, the addition of a higher concentration was needed when the assay was performed in yogurt (6 mg/liter) than during its aqueous phase (Fig. 3; Fig. S1). This can be attributed to viable bacteria present in the yogurt capable of taking up additional manganese during storage. Transcriptional profiling of BioP strains also identified high *mntH1* gene expression during storage (Fig. 4B), indicating continued transport activity in this period and thereby a potential for further bacterial manganese uptake during storage.

In the BioP strains, the expression level of the *mntH1* gene is exceptionally high, even surpassing most glycolytic genes. In comparison, an S. thermophilus starter culture strain and the less yeast growth-inhibiting L. paracasei strain C display a 25-fold-lower *mntH1* expression level. Consequently, an excessively high *mntH* expression level seems to be crucial for high bioprotective activity. The *mntH1* expression level of the BioP strains depends, however, on the stage of fermentation (Fig. 4B). A significant temporal increase in expression is observed, which could be an effect of a lowered manganese concentration caused by increases in cell quantity. This finding is in line with the manganese concentration-dependent *mntH* regulation mediated by MntR, previously observed in Bacillus subtilis (32). For the stationary-phase samples taken after 18 h of growth at 7°C, strain divergence in expression is observed, with expression remaining high in the L. paracasei strain B and less so for the L. rhamnosus strain A, possibly signifying species-dependent activity during cold storage. The level of *mntH1* expression was observed to increase during coculture of BioP strains compared to individual growth, possibly revealing a mutualistic or commensalistic relationship between the two organisms.

Deleting the highly transcribed *mntH1* gene in L. paracasei strain B proved this particular MntH1 transporter to be essential for the bioprotective phenotype. As the mechanism of bioprotection occurs by manganese scavenging, it follows that MntH1 is also the transporter responsible for manganese depletion. Although two additional MntH transporters and an ABC transport system (*sitABC*) exist in L. paracasei, there is no apparent sufficient regulatory response toward sustaining manganese uptake levels in the Δ*mntH1* mutant. Either the transport kinetics or the underlying transcriptional regulation of the alternative transport systems are inadequate to achieve the required manganese uptake level for inhibition of yeast growth. The relevance of the other transport systems could, however, be a function of manganese concentration and pH. The ABC transporter type has, for instance, been shown to display improved activity at neutral or alkaline pH, while MntH displayed improved activity in an acidic medium (21, 33). Previous studies of MntH in lactobacilli have provided ambiguous findings. In L. casei, deletion of two *mntH* transporters was required for reducing intracellular concentrations of manganese (34), while in another L. casei study, despite deletions in both *mntH* and *sitABC*, the growth rate was unaltered (35). The low manganese concentration tested in the former and latter study were, however, set at 0.1 mg/liter and 0.08 mg/liter, respectively, thus around 3 times higher than the concentration in milk. Possibly, these are too-high concentrations for a significant effect of *mntH1* deletion to materialize. Alternatively, significant strain variation in MntH1 activity exists, as already identified among the *Lactobacillus* strains A, B, and C examined in this study.

Variation in MntH1 activity could also explain our observation that not all selected lactobacilli (Fig. 6) harboring an *mntH* gene were equally proficient at inhibiting the growth of D. hansenii. Therefore, the presence of an *mntH* gene *per se* does not ensure good inhibition potential of spoilage organisms; rather, the expression level could be decisive. In the current experimental setting, the ability to take up manganese also requires the ability to grow in milk.

The main purpose of excessive manganese uptake appears to be the requirement for high intracellular manganese concentrations toward superoxide stress protection. As a simple and more efficient alternative exists in the manganese superoxide dismutase protein, additional reasons for high intracellular manganese concentrations may exist. It could be speculated whether excessive manganese scavenging as a competitive exclusion mechanism in itself could provide a fitness advantage in natural settings and thereby partially explain this characteristic among many lactobacilli.

In conclusion, a bacterial mechanism, originally identified as providing protection against oxidative stress, can, from this study, be coupled to facilitate another general bacterial mechanism, that of competitive exclusion. To our knowledge, such coupling in bacteria is without prior example. Moreover, it was shown that the principle of competitive exclusion, through the action of bioprotective strains, can be harnessed toward inhibiting the growth of unwanted spoilage organisms in a food source. The bioprotective activity of a strain in the present context, yogurt, can be defined as the sum of its ability to grow in milk, grow at the given fermentation temperature, and, not least, the expression of an *mntH* manganese transporter gene. Without the production of an antibiotic or other small molecule, competitive exclusion constitutes a mechanism that should be difficult for spoilage organisms to overcome by spontaneous mutation, making it an ideal mechanism for protection of food from a consumer, commercial, and regulatory perspective.

## MATERIALS AND METHODS

### Strains and growth conditions.

Strains used in this study are shown in Table 1. The yeast strains were streaked on yeast extract glucose chloramphenicol (YGC) plates (1 g/liter yeast extract [Merck], 20 g/liter d-glucose [Merck], and 0.1 g/liter chloramphenicol [Sigma-Aldrich]) and grown for 48 h at 25°C. Single colonies were inoculated to 25 ml YG media (1 g/liter yeast extract and 20 g/liter d-glucose) and grown overnight at 25°C with shaking. Afterward, glycerol was added to a final concentration of 15% (wt/vol), and the yeast cells were stored at −80°C until further use.

**TABLE 1:** Strains used in this study

Strain type and name	Comments/accession no.	Source
Yeasts		
*Torulaspora delbrueckii*		Chr. Hansen A/S
*Cryptococcus fragicola*		Chr. Hansen A/S
*Saccharomyces cerevisiae*		Chr. Hansen A/S
*Debaryomyces hansenii* strain 1		Chr. Hansen A/S
*Debaryomyces hansenii* strain 2		Chr. Hansen A/S
*Rhodotorula mucilaginosa*		Chr. Hansen A/S
Molds		
*Penicillium brevicompactum*		Chr. Hansen A/S
*Penicillium crustosum*		Chr. Hansen A/S
*Penicillium solitum*		Chr. Hansen A/S
Lactobacilli		
*L. rhamnosus* (strain A)	Part of BioP culture	Chr. Hansen A/S
*L. paracasei* (strain B)	Part of BioP culture	Chr. Hansen A/S
*L. paracasei* (strain C)		Chr. Hansen A/S
*L. rhamnosus* (strain 1)		Chr. Hansen A/S
*L. rhamnosus* (strain 2)		Chr. Hansen A/S
*L. rhamnosus* (strain 3)		Chr. Hansen A/S
*L. rhamnosus* (strain 4)	AZCQ00000000.1	LMG 6400
*L. salivarius*		Chr. Hansen A/S
*L. casei*		Chr. Hansen A/S
*L. paracasei*		Chr. Hansen A/S
*L. fermentum*		Chr. Hansen A/S
*L. sakei*	AZDN00000000	LMG 9468
*L. reuteri*	AZDD00000000	LMG 9213
*L. plantarum*		Chr. Hansen A/S
*L. brevis*		Chr. Hansen A/S
*L. kefiri*	AYYV00000000	DSM 20587
*L. alimentarius*		Chr. Hansen A/S
*Pediococcus acidilactici*	AEEG00000000	NCFB 2767
*L. delbrueckii* subsp. *bulgaricus*	*mntH* not present	Chr. Hansen A/S
*L. helveticus*	*mntH* not present	Chr. Hansen A/S
*L. gasseri*	*mntH* not present	DSM 20243
*ΔmntH1* mutant	*L. paracasei* strain B Δ*mntH1*	This study

The bacterial strains were streaked on MRS (Sigma-Aldrich) plates and incubated anaerobically at 37°C for 48 h. Single colonies were inoculated into 3 ml MRS medium and grown overnight at 37°C. Ten microliters of the overnight culture were added to 2 ml heat-treated milk containing 0.02% (vol/wt) commercial starter culture and a pH indicator. The acidification was performed at 43°C and stopped after ∼6 h when a pH of 4.5 was reached. The fermented milk was kept in the refrigerator upon further use.

### Yogurt production.

Reduced-fat (1.5% wt/vol) homogenized milk was heat treated at 90 ± 1°C for 20 min and cooled immediately. A commercial starter culture (S. thermophilus and L. delbrueckii subsp. *bulgaricus*) was inoculated at 0.02% (vol/wt) in 3-liter buckets. One bucket was inoculated with bioprotective culture (+BioP) in a total concentration of 100 U/ton, and one bucket was used as a reference (REF) and only inoculated with the starter culture. All buckets were incubated in a water bath at 43°C and fermented at these conditions until a pH of 4.60 was reached. At this time point, the fermented milk products were divided into 200-ml bottles and cooled down.

### Aqueous phase production and manganese concentration determination.

The fermented milk product was centrifuged (10 min at 5,000 rpm), and the supernatant (AQ) was sterile filtered. The manganese concentration of the AQ was determined by inductively coupled plasma mass spectrometry at Eurofins Steins Laboratorium A/S.

### Assay to detect bioactivity in AQ against yeasts.

The AQ of reference and BioP were transferred to a sterile 96-well plate (150 μl in each well), and dilutions were performed or different concentrations of metals were added (6 mg/liter to 6 ng/liter of manganese, 0.3 mg/liter of iron, 0.1 mg/liter of copper, 4.2 mg/liter of zinc, and 60 mg/liter magnesium).

Washed L. paracasei (wild-type strain B and Δ*mntH1*) cultures were added to 2 ml milk and grown for ∼18 h at 37°C until pH 4.5 was reached. The fermented milk was centrifuged (10 min at 5,000 rpm), and the supernatant (AQ) was transferred to a sterile 96-well plate (150 μl in each well).

The different yeasts (Table 1) were taken from the glycerol stock and diluted in MilliQ H_2_O to inoculate with ∼20 cells per well. The plates were incubated at 17°C for several days, and the yeast growth was determined by measuring the absorbance at 600 nm.

### Assay to detect bioactivity of lactobacilli against yeast in fermented milk.

Lactobacilli were selected from the Chr. Hansen A/S strain collection. If available, species type strains were selected, while strains from the remaining species were selected based on the availability of the corresponding genome sequence in an otherwise unbiased selection process. One hundred fifty microliters of the fermented milk were transferred to individual wells in a 96-well plate. Manganese (6 mg/liter) was added to half of the samples, and the wells were inoculated with about 20 cells of the respective yeast. After 4 days of incubation at 17°C, a dilution row was spotted on selective YGC agar plates to analyze the yeast growth. In some cases, the growth was enumerated by optical inspection, where a value of 0 was given for no growth, a value of 1 for 1 to 2 colonies, a value of 2 for 2 to 10 colonies, a value of 3 for 10 to 30, a value of 4 for 30 to 70, and a value of 5 for confluent growth.

### Assay to detect bioactivity in yogurt against molds.

The fermented milk products were divided into 200-ml bottles and cooled down. Different manganese concentrations (6 ng/liter to 6 mg/liter) were added to fermented milk products with BioP. All the fermented milk samples were heated to a temperature of 40°C and supplemented with 40 ml of a 5% sterile agar solution that had been melted and cooled down to 60°C. This solution of fermented milk and agar was then poured into sterile petri dishes and the plates were dried in an LAF bench for 30 min. Three target contaminants, Penicillium brevicompactum, Penicillium crustosum, and Penicillium solitum, were added in concentrations of 500 spores/spot. The plates were incubated at 22 ± 1°C for 8 days before assessment of mold growth.

### Isolation and processing of RNA.

Initially, three independent precultures of each strain were cultivated anaerobically overnight at 37°C. Cultures were washed twice in 0.9% saline solution and inoculated in milk, which, in this case, was skim milk powder reconstituted (9.5%) in distilled water. Strain A was inoculated to an optical density at 600 nm (OD_600_) corresponding to 0.1, strain B to 0.5, strain C to 0.3, and the combined BioP culture to 0.1. The differences in inoculation level were chosen based on preliminary acidification rates and a growth rate proxy, with an inverse relationship between acidification rate and selected inoculation level. Prior to inoculation, lactic acid was added to reduce pH to 5.5 to simulate the acidification normally provided by the starter culture. Milk fermentation was performed at 37°C in 2-ml deep-well plates covered by foil, and proceeded either for 4 h and 6 h before harvest or for 6 h at 37°C and followed by 18 h at 7°C. Growth was performed in triplicate samples for each strain, and not all strains were subjected to all conditions. Cell harvest was performed by mixing a 1:2 volume of milk fermentate (2 ml) to RNAprotect bacteria reagent (Qiagen) (4 ml) followed by a procedure for separation of cells from milk fermentate, adapted from Derzelle et al. (36). A 2-ml volume of 1 M sodium citrate solution and 0.78 ml saline solution (0.145 M sodium chloride, 0.016 M sodium β-glycerophosphate, 0.1% Tween 80, pH 7) were added and, after 5 min, centrifuged at 10,000 × *g* for 2 min. The resulting cell pellet was washed with cold phosphate buffer (5 mM sodium phosphate, 1 mM EDTA, pH 7) and centrifuged as before, and the supernatant was discarded and the cell pellet was frozen at −80°C. The cell pellet was dissolved in Tris-EDTA buffer containing lysozyme (15 mg/ml), proteinase K (1.3 mg/ml), and mutanolysin (50 U) and was shaken at 1,400 rpm for 10 min at 37°C. The subsequent RNA extraction procedure was performed with the RNeasy protect bacteria minikit (Qiagen) per the manufacturer’s instructions, including removal of DNA with DNase I. The quality of total RNA was evaluated using a Bioanalyzer 2100 (Agilent). Depletion of rRNA, library preparation, and sequencing (Illumina 50-bp single-end sequencing) was performed at Keygene NV, The Netherlands.

### RNA-seq data analysis.

Obtained raw reads, 15 to 45 million per sample, were trimmed with Trimmomatic (37) using default parameters, and mapping was performed using CLC Genomics Workbench v10 (Qiagen) with the following parameters: mismatch cost, 2; insertion cost, 3; deletion cost, 3; length fraction, 0.8; similarity fraction, 0.9; and strand-specific local alignment and maximum number of hits for a read of 10. For reads with 2 to 10 hits, reads were randomly assigned a gene. For BioP culture samples, mapping was performed for both genomes simultaneously and to the relevant individual genome for strain A, B, and C samples. Strain C reads were mapped to the strain B genome, as they are identical species. Following mapping, total gene counts were extracted. Within the SARTools (38) framework, the DESeq2 R package (39) was applied for normalization of sample gene counts using the median ratio method and subsequent estimation of differential expression. For comparison of *mntH* gene counts, it was necessary to compare gene counts between samples of different species that harbor distinct genes and therefore cannot immediately be normalized together using DESeq2. Therefore, an additional normalization between sample types containing different species compositions (BioP, strain A, strain B, or strain C) was performed on within-species normalized gene counts applying transcripts per kilobase million (TPM) normalization. For BioP culture samples, additional TPM normalization was performed for the sample as a whole (sample normalized), but also individually for strain A and strain B (strain normalized). This gives different gene count distributions, as more counts were associated overall with strain B over strain A for the 6-h samples.

### Generation of the Δ*mntH1* mutant.

Clean deletion of the entire Δ*mntH1* in L. paracasei strain B was performed by a combination of homologous recombination and CRISPR-Cas9 counter selection (Text S1), as previously described (40). Briefly, 500-bp homology arms flanking *mntH1* were employed for homologous recombination, while a targeting CRISPR-Cas9 nuclease was utilized for selection of Δ*mntH1* deletion mutants. Transformation of L. paracasei was adapted from Song et al. (41), and to improve transformation efficiency, a procedure for obtaining nonmethylated plasmids was employed (42). Once a correct mutant was isolated and sequence verified, the plasmids were cured through nonselective growth cycles. Oligonucleotides and plasmids used in this study are shown in Table 2 and 3, respectively.

**TABLE 2:** Oligonucleotides used in this study

Name	Sequence
oJV2	GGCCGCATGTTTTGGGACCATTCAAAACAGCATAGCTCTAAAACTTGTTCTGATCGTAGCTATCCTCGAT
oJV3	CTGTAATTTGTTTAATTGCCATTTCAATT
oJV4	GGAACTACAAAATAAATTATAAGGAGGC
oJV5	AATAACTCTCCCCTTTCG
oJV6	TTTGTTTGAATCTTTGGCC
oJV7	TTTTGCTCACATGTTCTTTC
oJV8	CTGCTTTTTGGCTATCAATC
oJV9	CCGCTTCGGTTTGAGCTTTGAT
oJV10	TGACATGCTGCTTTTTGGCCAT
oJV11	AGCGGCTTTACTAGGGAAACTGA
oEFB0072	GGAGCTGTAATATAAAAACCTTCTTC
oEFB0106	AAGCTTTCTTTGAACCAAAATTAG
oEFB0107	GAAGAAGGTTTTTATATTACAGCTCCGTGACTTTTTAACAATAACGGCAATTC
oEFB0108	CTAATTTTGGTTCAAAGAAAGCTTTCATTGTTCGTCAACATCTGCCTTCG
oEFB0055	CAACATCTTCGCTGCAAAGC
oEFB0056	CTCTATTCAGGAATTGTCAG

**TABLE 3:** Plasmids used in this study

Plasmid	Description	Resistance to^*a*^:	Source or reference
pCB578	*E. coli*-lactobacilli shuttle vector containing SpCas9, tracrRNA, and a repeat-spacer-repeat array	Erm	40
pJV114	Shuttle vector with pCB578 base containing a spacer targeting *mntH1*	Erm	This work
pCB591	*E. coli*-lactobacilli shuttle vector for homologous recombination template cloning	Amp (*E. coli*) Cm (*Lactobacillus*)	40
pJV88	Shuttle vector with pCB591 base containing the *mntH1* gene and 500-bp homology arms on either side	Amp (*E. coli*) Cm (*Lactobacillus*)	This work
pJV91	Shuttle vector with pCB591 base containing 500-bp homology arms on either side of *mntH1* that serves as a recombination template to generate a clean deletion	Amp (*E. coli*) Cm (*Lactobacillus*)	This work
pNZ8148	Broad-host-range LAB expression vector containing NisA promoter and pepN terminator	Cm	Mobitech
pEFB021	pNZ8148 in which NisA promoter has been replaced by the 207-bp native *mntH1* promoter followed by *mntH1*	Cm	This work

a Erm, erythromycin; Amp, ampicillin; Cm, chloramphenicol.

### Statistical analysis.

Statistical analyses were conducted using Graph Pad Prism 8.2.0 (GraphPad Software, Inc., San Diego, CA). When applying one- or two-way ANOVA analyses, multiple comparisons were corrected for using Tukey’s statistical hypothesis testing. All relevant statistical information is reported along with *P* and *n* values.

### Data availability.

Transcriptomic data are available from the NCBI Sequence Read Archive (SRA) repository under BioProject accession no. PRJNA602536. Strains will be available for research purposes upon request.

## Supplementary Material

Supplemental file 1
