# Supplementary material for: Competitive Exclusion Is a Major Bioprotective Mechanism of Lactobacilli against Fungal Spoilage in Fermented Milk Products
Source: Appl Environ Microbiol. 2020 Mar 18;86(7):e02312-19. doi: 10.1128/AEM.02312-19 (PMC7082583; doi:10.1128/AEM.02312-19)
Supplement: Supplemental file 1 [file AEM.02312-19-s0001.pdf]

Supplemental material to:

Competitive exclusion is a major bioprotective mechanism of lactobacilli against fungal spoilage in fermented milk products

Solvej Siedler[a*#], Martin Holm Rau[a*], Susanne Bidstrup[a], Justin M. Vento[b],
Stina D. Aunsbjerg Nielsen[c], Elleke F. Bosma[a], Laura F. McNair[d], Chase L. Beisel[b,e,f], Ana Rute Neves[a]

[a] Discovery, R&D, Chr. Hansen A/S, Bøge Allé 10-12, 2970 Hørsholm, Denmark

[b] Department of Chemical and Biomolecular Engineering, North Carolina State University, Raleigh, NC 27695, USA

[c] Global Application, Chr. Hansen A/S, Bøge Allé 10-12, 2970 Hørsholm, Denmark

[d] Department of Drug Design and Pharmacology, University of Copenhagen, Universitetsparken 2, Copenhagen, Denmark

[e] Helmholtz Institute for RNA-based Infection Research (HIRI), Helmholtz Center for Infection Research, Würzburg, Germany

[f] Medical Faculty, University of Würzburg, Würzburg, Germany

Running Head: Competitive exclusion by lactobacilli

#Address correspondence to S.S.

[*] S. S. and M. H. R. contributed equally to this work

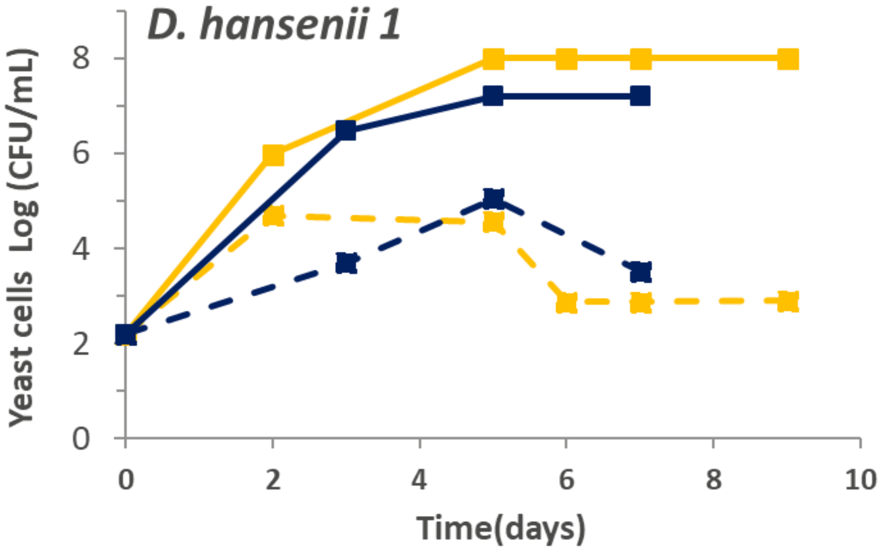

Figure S1: Comparison of *D. hansenii* Strain 1 growth in yogurt (yellow symbols) and its aqueous phase (blue symbols) with (dotted line) and without (solid line) bioprotective culture. *D. hansenii* was inoculated (100 cells/ml) and growth was followed by spotting dilution rows on selective YGC plates to count the yeast colonies at the indicated time points after inoculation. CFU, colony forming units

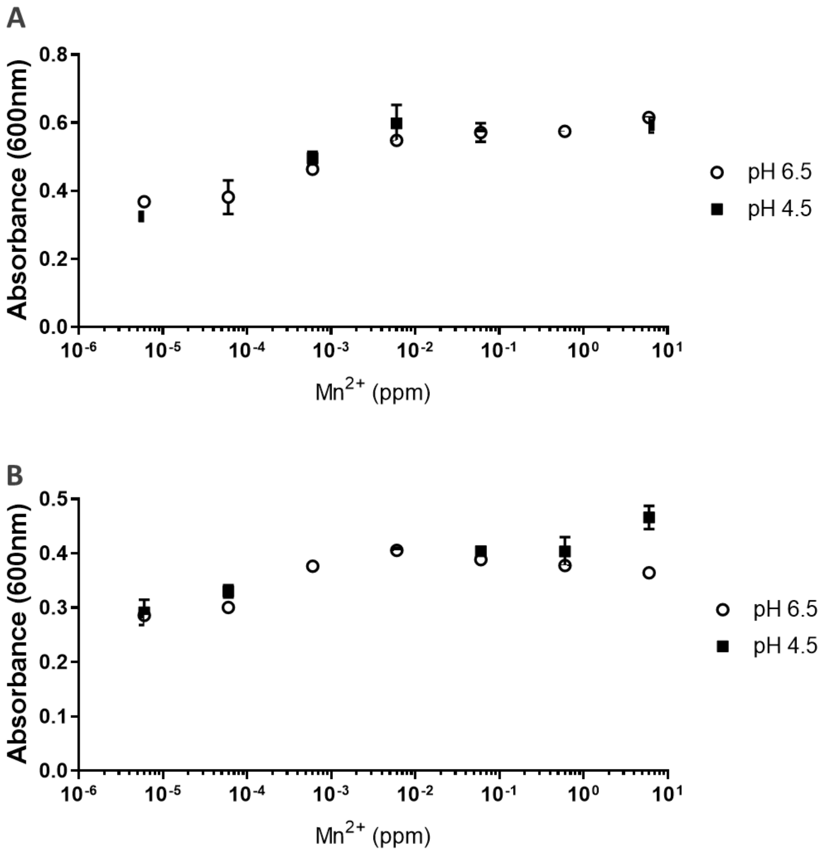

Figure S2: Effect of manganese concentration on yeast growth. Absorbance measurements of *D. hansenii* Strain 1 (**A**) and Strain 2 (**B**) after growth in a chemically defined medium[1] (CDM) as a function of manganese concentration. Two initial CDM pH setpoints were tested.

[1] Chemically defined medium. The CDM contains (per liter of demineralized water): 20 g glucose, 1 g Na-acetate, 0.6 g NH_4-citrate, 5 g KH_2PO_4, 1 g K_2HPO_4, 0.24 g L-alanine, 0.5 g L-arginine, 0.125 g L-asparagine, 0.42 g L-aspartic acid, 0.13 g L-cysteine, 0.5 g L-glutamic acid, 0.175 g L-glycine, 0.15 g L-histidine, 0.21 g L-isoleucine, 0.475 g L-leucine, 0.44 g L-lysine, 0.125 g L-methionine, 0.275 g L-phenylalanine, 0.675 g L-proline, 0.34 g L-serine, 0.225 g L-threonine, 0.05 g L-tryptophan, 0.25 g L-tyrosine, 0.325 g L-valine, 0.01 g of each of the four nucleobases adenine, guanine, uracil and xanthine, 0.2 g $MgCl_2 \cdot 6\ H_20$, 0.05 g $CaCl_2 \cdot 2H_20$, 5 mg $Zn_2SO_4 \cdot 7H_20$, 2.5 mg $CoSO_4 \cdot 7H_20$, 2.5 mg $CuSO_4 \cdot 5H_2O$, 2.5 mg $(NH_4)_6Mo_7O_{24} \cdot 4H_2O$, 3 mg $FeCl_3 \cdot 6H_2O$, 5 mg $FeCl_2 \cdot 4H_2O$, 0.1 % Tween 80, 0.5 g ascorbic acid, 2 mg pyridoxine-HCl, 5 mg pyridoxamine-HCl, 10 mg p-aminobenzoic acid, 1 mg nicotinic acid, 1 mg Ca-DL-panthothenate, 1 mg thiamine, 1 mg lipoic acid, 1 mg riboflavin, 2.5 mg biotin, 1 mg folic acid and has a final pH at 5.6.

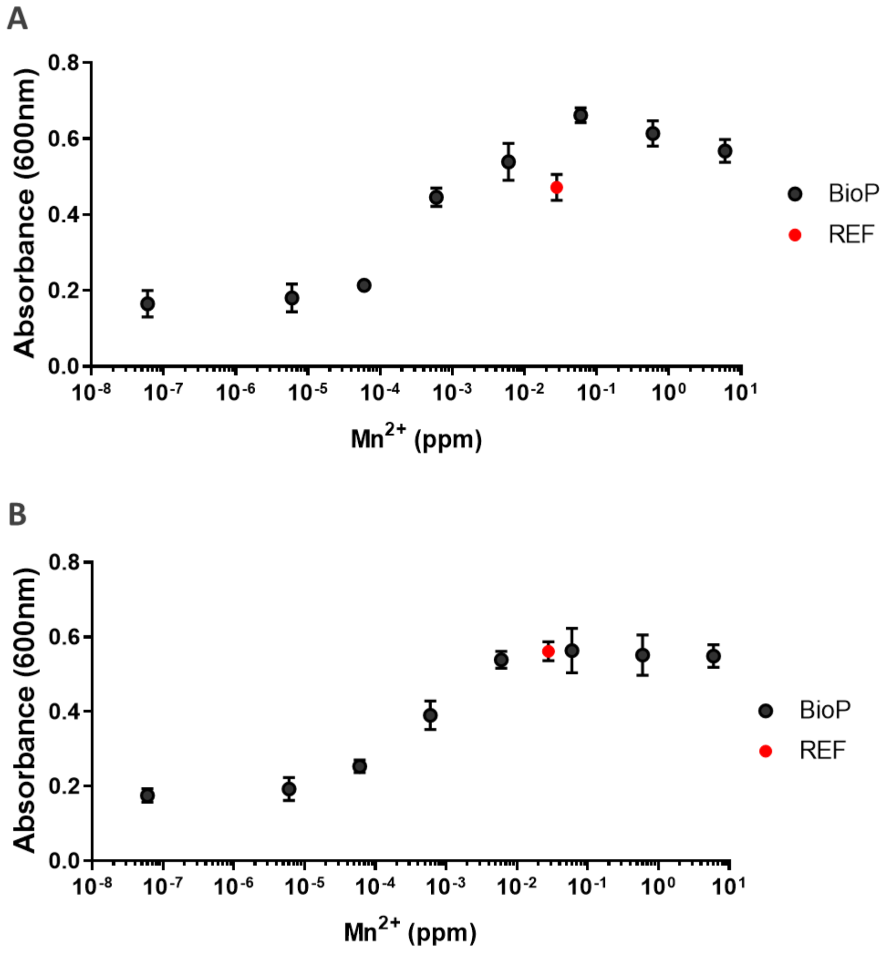

Figure S3: Effect of manganese addition on yeast growth in BioP AQ phase. Absorbance measurements of *D. hansenii* Strain 1 (**A**) and Strain 2 (**B**) after 7 days of incubation at 17°C in AQ phase. Influence of Mn^{2+} addition to BioP AQ phase (black), compared to reference (REF) yoghurt aqueous phase (red) without any added manganese. Average and standard deviation of technical replicates n=6 (**A**) and n=3 (**B**) are shown.

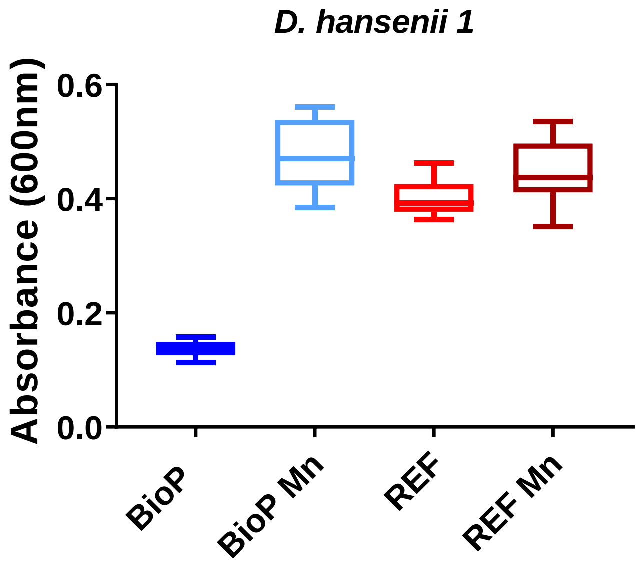

Figure S4: *D. hansenii* Strain 1 growth in REF AQ and BioP AQ without (dark blue) and with (light blue) the addition of 0.6 mg/L manganese and in REF AQ without (light red) and with (dark red) manganese addition after 5 days of incubation at 17°C. Box and whiskers of 6-9 replicates are shown.

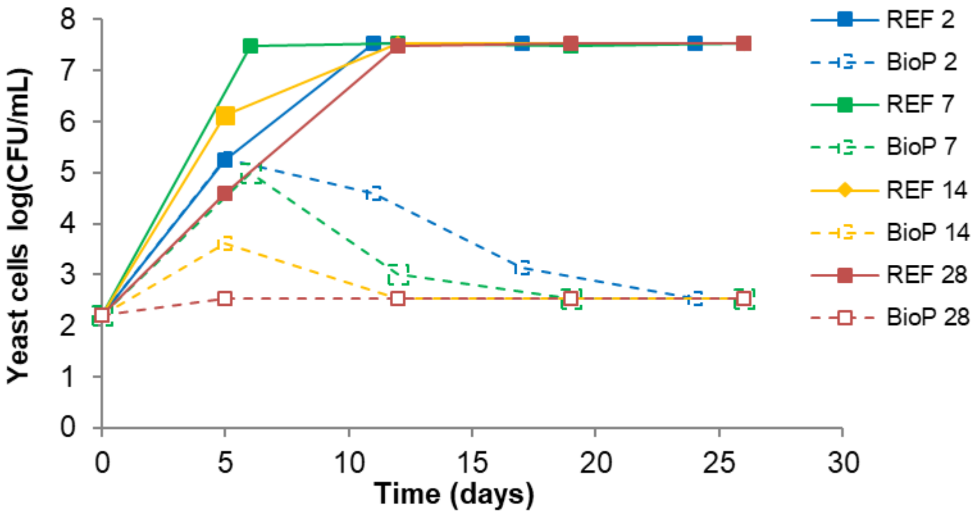

Figure S5: Change in bioactivity of BioP containing yogurt after storage, *D. hansenii* was inoculated (100 cells/ml) at different time points after yogurt production (blue, 2 days; green, 7 days; yellow, 14 days and red 28 days). Yogurt was produced with (dotted line) and without (solid line) BioP. The inoculated yogurt was incubated at 7°C and *D. hansenii* growth was followed by spotting dilution rows on selective YGC plates to count the yeast colonies at the indicated time points after inoculation.

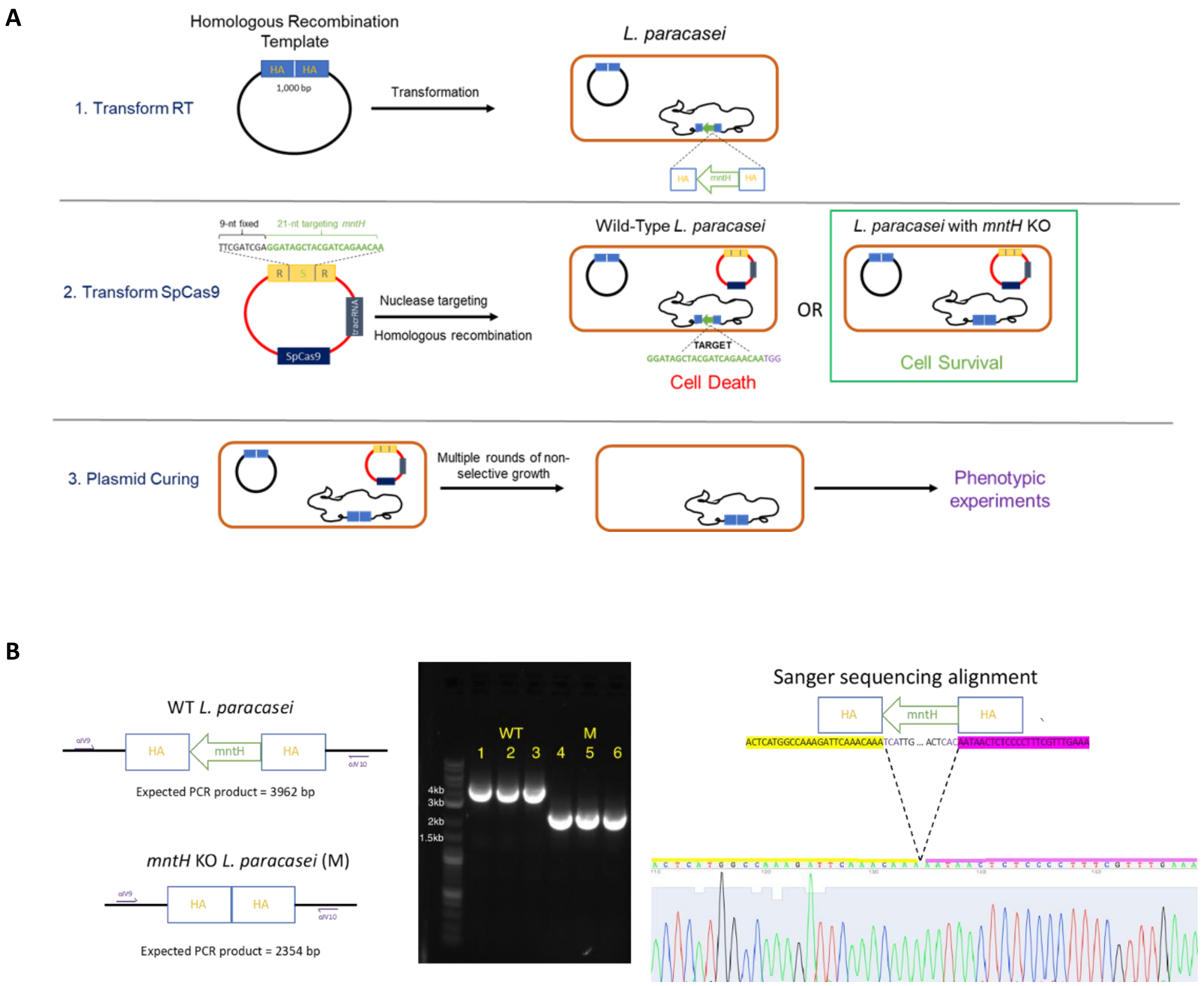

Figure S6: CRISPR-Cas9 genome editing in *L. paracasei* to delete the *mntH*1 gene. (**A**) Scheme to generate a clean knockout of the *mntH*1 gene using a homologous recombination (HR) template and CRISPR-Cas9 shuttle vectors. First, a shuttle vector harboring a recombineering template containing 500-bp homology arms on either side of the *mntH*1 coding region was transformed into *L. paracasei*. Then, another shuttle vector encoding SpCas9, a tracrRNA, and a single-spacer CRISPR array targeting *mntH*1 was transformed into the HR template strain to remove any unedited cells. Plasmids were cured from the mutant strains by non-selective growth in MRS media and on agar plates before the resulting mutant was subjected to phenotypic experiments. **(B**) Validation of the *mntH*1 deletion via gel electrophoresis and Sanger sequencing. (Left) after transforming the targeting CRISPR-Cas9 shuttle vector, colonies were screened by cPCR that amplifies from the genome of *L. paracasei* but not the plasmid with the HR template. (Center) samples 1-3 on the gel show the PCR product obtained from the WT genome while Samples 4-6 show the PCR product obtained from a mutant (M) strain. (Right) the PCR product from the mutant strain was then subjected to Sanger sequencing to confirm that the start codon through the stop codon of *mntH*1 was successfully deleted.

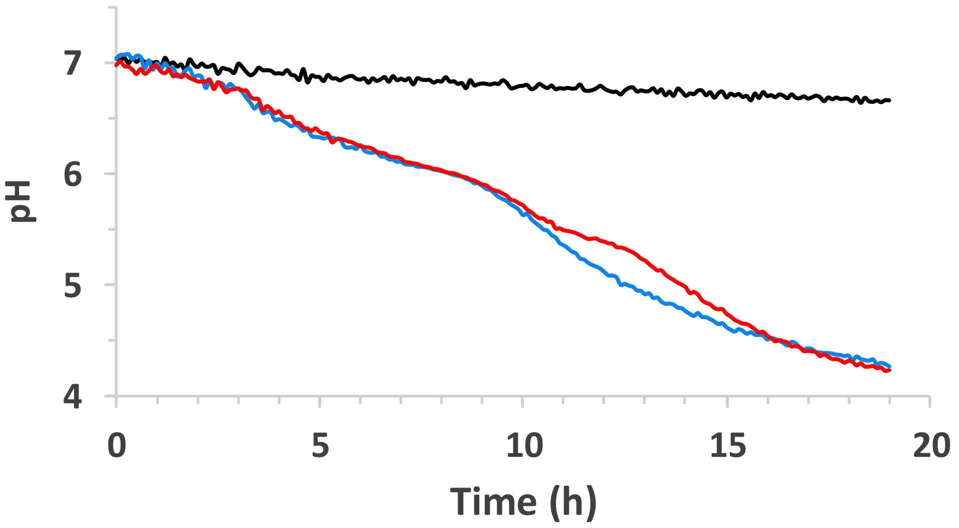

Figure S7: Acidification profile of BioP Strain B (blue) and its Δ*mntH*1 mutant (red) and a control of milk, which had not been inoculated (black). Averages of 3 replicates are shown.

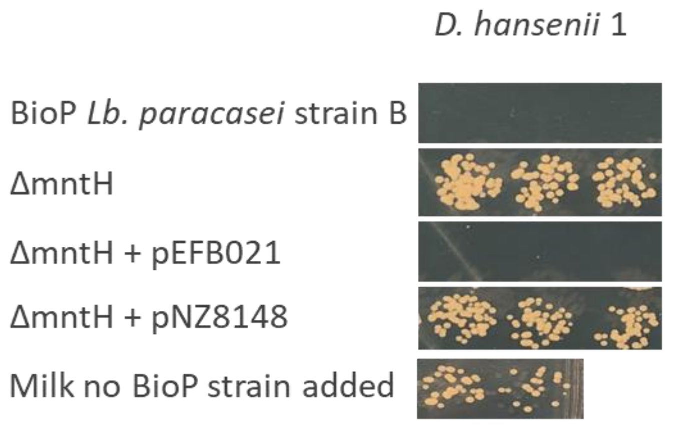

Figure S8: Influence of complementation of the Δ*mntH*1 mutant on its bioactivity. Milk was fermented by BioP *Lb. paracasei* strain B, its Δ*mntH*1 mutant and the Δ*mntH*1 mutant harboring the empty plasmid (Δ*mntH*1 + pNZ8148) or a plasmid containing the *mntH*1 gene under its own promoter (Δ*mntH*1 + pEFB021). Afterwards, *D. hansenii* 1 was inoculated (20 cell) and after 4 days of incubation at 17°C a dilution row was spotted on selective YGC agar plates to analyze the yeast growth.

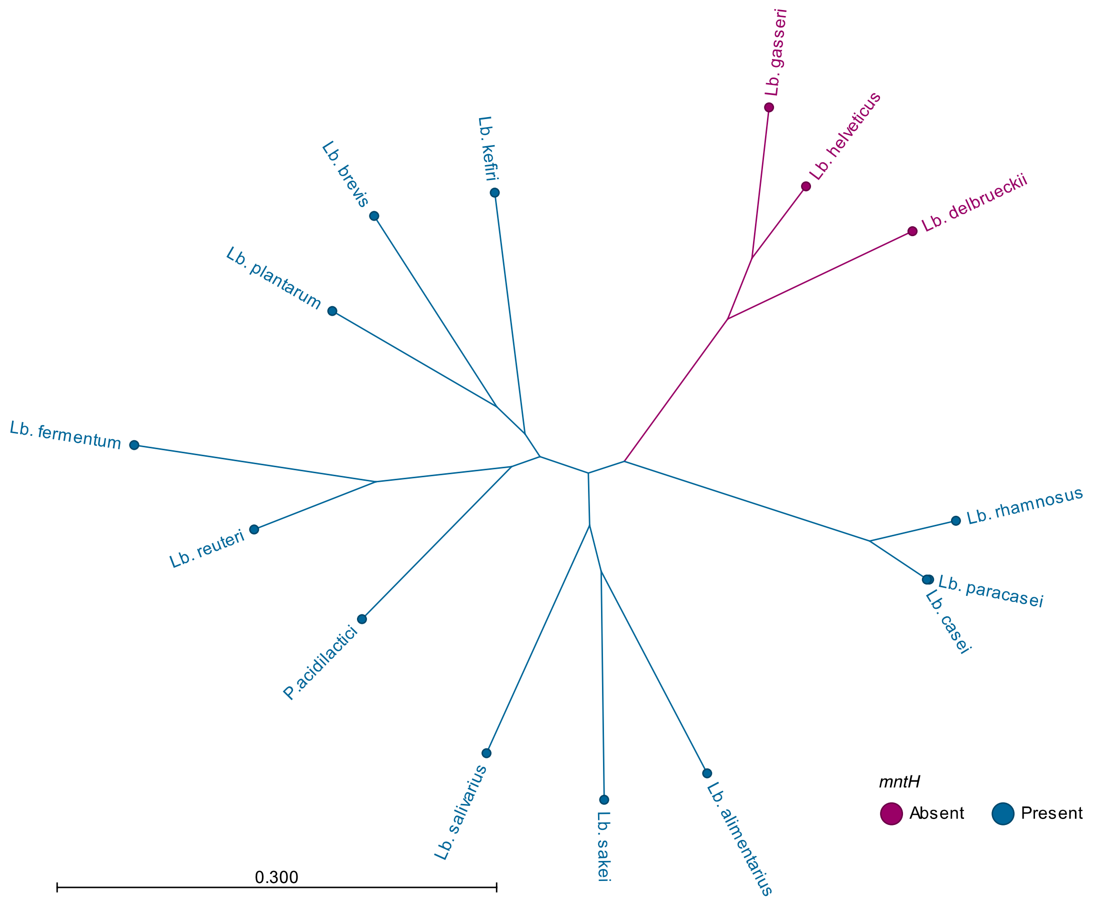

Figure S9: Phylogenetic tree of the assayed lactobacilli with genomic *mntH* gene absence or presence denoted by red or blue color, respectively. Workflow of phylogenetic inference is described in Text S1.

Table S1: Sample-normalized and strain-normalized counts for all manganese transport-related genes and top10 expressed genes

ID	Function	BioP 4h	BioP 6 h	BioP 24 h	Strain A and Strain B[a]	Strain A[a]	Strain B[a]	Strain C[a,b]
Strain_A_1992	Manganese transport protein MntH	18	26	3	72	86	NA	NA
Strain_A_2430	Manganese transport protein MntH	948	6551	1028	18053	5170	NA	NA
Strain_B_31	Manganese transport protein MntH	241	44	249	69	NA	95	178
Strain_B_959	Manganese transport protein MntH	25	26	36	41	NA	43	67
Strain_B_2310	Manganese transport protein MntH	1208	9838	8543	15442	NA	10907	555
Strain_A_2438	Manganese ABC transporter protein SitA	335	187	21	516	250	NA	NA
Strain_A_2439	Manganese ABC transporter protein SitD	318	184	19	506	228	NA	NA
Strain_A_2440	Manganese ABC transporter protein SitB	321	178	13	491	191	NA	NA
Strain_B_2320	Manganese ABC transporter protein SitA	227	1241	35	1949	NA	302	56
Strain_B_2321	Manganese ABC transporter protein SitD	179	884	26	1388	NA	211	42
Strain_B_2322	Manganese ABC transporter protein SitB	191	928	30	1456	NA	220	40
Strain_A_976	Glyceraldehyde-3-phosphate dehydrogenase	10275	10375	22218	28582	25938	NA	NA
Strain_A_487	NADH peroxidase Npx	5244	7044	1722	19406	7175	NA	NA
Strain_A_1363	Translation elongation factor Tu	8191	6603	1954	18195	13773	NA	NA
Strain_A_2430	Manganese transport protein MntH	948	6551	1028	18053	5170	NA	NA
Strain_A_2432	FIG00743426: hypothetical protein	2783	6743	5038	18577	11550	NA	NA
Strain_A_1651	Oligopeptide ABC transporter OppA	3089	6151	301	16947	21545	NA	NA
Strain_A_2541	L-lactate dehydrogenase	3748	5159	1602	14214	9272	NA	NA
Strain_A_979	Enolase	3479	3666	5322	10103	6352	NA	NA
Strain_A_1395	Pyruvate kinase	1546	3197	2751	8811	5227	NA	NA
Strain_A_1412	DNA-binding protein HBsu	4064	3253	2313	8965	10756	NA	NA
Strain_B_1921	Glyceraldehyde-3-phosphate dehydrogenase	15383	23054	42198	36189	NA	38272	NA
Strain_B_1282	Oligopeptide ABC transporter OppA	3037	14088	2287	22116	NA	24149	NA
Strain_B_2312	FIG00743426: hypothetical protein	3399	13596	23936	21342	NA	22030	NA
Strain_B_2635	Molecular chaperone (small heat shock protein)	20483	11961	13010	18775	NA	30622	NA
Strain_B_1560	Translation elongation factor Tu	6499	10528	4888	16525	NA	17731	NA
Strain_B_1513	DNA-binding protein HBsu	7237	10615	7568	16662	NA	16710	NA
Strain_B_2310	Manganese transport protein MntH	1208	9838	8543	15442	NA	10907	NA
Strain_B_2434	L-lactate dehydrogenase	5329	7977	11579	12521	NA	13460	NA
Strain_B_1918	Enolase	7896	5590	9580	8774	NA	9875	NA
Strain_B_1769	Cold shock protein CspA	2223	5054	7622	7933	NA	5753	NA

[a] Strain-normalized counts, not sample-normalized counts

[b] *Lb. paracasei* Strain C reads mapped to *Lb. paracasei* Strain B genes

Text S1

Phylogeny of Lactobacillus strains

The genomes of the selected *Lactobacillus* strains were annotated using RAST (1) and core genes identified using Roary (2) with a minimum of 80 % identity. The core genes were aligned using prank (3), poorly aligned positions eliminated using gblocks (4) and the Maximum-Likelihood phylogeny inferred using RAxML-NG (5) with the GTR GAMMA (GTR+F0+G) model, 20 random starting trees and 100 bootstrap replicates. The phylogenetic tree was constructed using CLC Genomics Workbench v11 (CLC Bio A/S). Presence of genome encoded MntH proteins was identified using tblastn with BioP MntH and prokaryotic TCDB (6) 2.A.55 Nramp family transporters as queries.

Plasmid generation

The Cas9 targeting plasmid pJV114 was generated from *E. coli*-lactobacilli shuttle vector pCB578 containing SpCas9, a tracrRNA, and a repeat-spacer-repeat array (7). To clone a new spacer targeting *mntH*1, pCB578 was digested with PvuI and NotI enzymes, and oligos oJV1-2 containing a 21-nt target-derived spacer and the 3' repeat were phosphorylated with T4 PNK and annealed together before being ligated into this digested backbone with Instant Sticky-end Ligase Master Mix (NEB CN# M0370S). The resulting spacer includes 9 fixed nts at the 5' end that do not participate in targeting, resulting in a 30-nt spacer that is processed as part of CRISPR RNA biogenesis. The ligation mix was transformed into NEB 5-alpha Competent *E. coli* (NEB CN #C2987I) and plated on LB agar containing 300 µg mL^{-1} erythromycin. Colonies were screened with cPCR primers oJV3-4 and Sanger sequenced with oJV4 to confirm the correct spacer sequence.

The homologous recombination template plasmid pJV91 was generated in two steps beginning with the *E. coli*-lactobacilli shuttle vector pCB591 (7). First, the *mntH*1 gene was amplified from the *Lb. paracasei* genome along with 500-bp homology arms on either side, and the PCR fragment was inserted into pCB591 using the Gibson assembly kit (NEB CN# E2611S) to generate pJV88. Then,

the *mntH*1 gene was deleted from pJV88 using the Q5 site-directed mutagenesis kit (NEB CN# E0554S) following the manufacturer's instructions along with primers oJV5-6 to generate pJV91. NEB 5-alpha Competent *E. coli* cells were used for both cloning steps, and primers oJV7-8 were used for cPCR of *E. coli* colonies. Gel electrophoresis and Sanger sequencing with oJV7-8 were used to confirm the correct clones.

The *mntH1*-reconstitution plasmid pEFB021 was generated via 2-part Gibson assembly. The backbone of expression vector pNZ8148 without the *NisA* promoter but including the *PepN* terminator was amplified using oEFB0072+0106. For pEFB021, the native 207-bp *mntH1* promoter followed by the *mntH1* gene were amplified from the *Lb. paracasei* genome using oEFB0107+0108, which contained overhangs with oEFB0072+0106. The two fragments were assembled using the NEBuilder HiFi DNA Assembly Master Mix (NEB #E2621S). A clean-up step was performed using the Monarch PCR & DNA Cleanup Kit (NEB #T103S), in which elution was performed with 20 µL MiliQ water. 5 µL of the cleaned-up DNA was then transformed to electrocompetent *L. lactis* MG1363 (8) and plated on GM17-agar containing 10 µ/mL chloramphenicol. Colonies were screened by PCR with primers oEFB0055+0056 and Sanger-sequenced with the same primers to confirm correct constructs.

Transformation of plasmids

The transformation protocol used for mutant generation in *Lb. paracasei* was adapted from Song et. al (9). Briefly, overnight cultures were back-diluted in 50 mL of MRS supplemented with 1% glycine and grown until OD_{600} reached 0.6-0.8. Then, the cells were harvested before being washed twice with ice-cold 10% glycerol. Finally, cells were resuspended in 500 µL of ice-cold 10% glycerol. For all transformations, 100 µL of this suspension and 1 µg of plasmid DNA was added to a 2-mm gap cuvette and transformed at 2.0 kV, 200 Ω resistance, and 25 µF capacitance. Following electroporation, 900 µL of MRS supplemented with 500 mM sucrose, 2 mM $CaCl_2$, and 20 mM $MgCl_2$ was added to the cuvette and transferred to a sterile tube and incubated at 37 °C without shaking for 3 hours. Then, 250 µL of this recovery was plated on MRS agar containing any necessary antibiotics,

and the plates were incubated at 37 °C for 3 days before counting colonies. Chloramphenicol and erythromycin concentrations were both 10 μg mL^{-1} in MRS liquid media and agar.

Genome editing

To perform the genomic edit, the homologous recombination template plasmid pJV91 was isolated from the non-methylated *E. coli* strain EC135 to improve transformation efficiency (7, 10) and transformed into *Lb. paracasei*. Colonies containing pJV91 were selected for by plating on MRS agar supplemented with chloramphenicol. A single colony was inoculated in liquid culture and cells were made electrocompetent using the previously described methods. Then, the targeting CRISPR-Cas9 plasmid pJV114 was passaged through EC135 and transformed into the homologous recombination template strain. The recovered cells were plated on MRS agar supplemented with erythromycin to select only for the CRISPR-Cas9 plasmid, and a significant increase in CFU was observed when transforming pJV114 into the recombination template strain compared to the WT strain. Colonies were screened using cPCR with primers oJV9-10 that would only amplify from the *Lb. paracasei* genome, and the PCR products were subjected to gel electrophoresis and Sanger sequencing with oJV11 to validate the genomic deletion (**Fig S6**). Both plasmids were cured from the mutant *Lb. paracasei* strain by cycling between culturing a colony in MRS media and plating the culture on MRS agar, both without antibiotics. After each round of plating, single colonies were picked and grown in MRS media and replica plated on MRS agar supplemented with either chloramphenicol or erythromycin. This cycle was repeated until a mutant strain was sensitive to either antibiotic. Finally, the resulting mutant strain was subjected to the phenotypic experiments.

Supplemental references:

1. Overbeek R, Olson R, Pusch GD, Olsen GJ, Davis JJ, Disz T, Edwards RA, Gerdes S, Parrello B, Shukla M, Vonstein V, Wattam AR, Xia F, Stevens R. 2014. The SEED and the Rapid Annotation of microbial genomes using Subsystems Technology (RAST). Nucleic Acids Res 42:D206-14.

2. Page AJ, Cummins CA, Hunt M, Wong VK, Reuter S, Holden MTG, Fookes M, Falush D, Keane JA, Parkhill J. 2015. Roary: rapid large-scale prokaryote pan genome analysis. Bioinformatics 31:3691–3693.

3. Löytynoja A, Goldman N. 2005. An algorithm for progressive multiple alignment of sequences with insertions. Proc Natl Acad Sci U S A 102:10557–62.

4. Talavera G, Castresana J, Kjer K, Page R, Sullivan J. 2007. Improvement of Phylogenies after Removing Divergent and Ambiguously Aligned Blocks from Protein Sequence Alignments. Syst Biol 56:564–577.

5. Kozlov AM, Darriba D, Flouri T, Morel B, Stamatakis A. 2019. RAxML-NG: a fast, scalable and user-friendly tool for maximum likelihood phylogenetic inference. Bioinformatics 35:4453–4455.

6. Saier MH, Reddy VS, Tsu B V., Ahmed MS, Li C, Moreno-Hagelsieb G. 2016. The Transporter Classification Database (TCDB): recent advances. Nucleic Acids Res 44:D372–D379.

7. Leenay RT, Vento JM, Shah M, Martino ME, Leulier F, Beisel CL. 2019. Genome Editing with CRISPR-Cas9 in *Lactobacillus plantarum* Revealed That Editing Outcomes Can Vary Across Strains and Between Methods. Biotechnol J 14:1700583.

8. Nickoloff JA, Holo H, Nes IF. 2003. Transformation of *Lactococcus* by Electroporation, p. 195–200. *In* Electroporation Protocols for Microorganisms. Humana Press.

9. Song X, Huang H, Xiong Z, Ai L, Yang S. 2017. CRISPR-Cas9D10A Nickase-Assisted Genome Editing in *Lactobacillus casei.* Appl Environ Microbiol 83:e01259-17.

10. Zhang G, Wang W, Deng A, Sun Z, Zhang Y, Liang Y, Che Y, Wen T. 2012. A Mimicking-of-DNA-Methylation-Patterns Pipeline for Overcoming the Restriction Barrier of Bacteria. PLoS Genet 8:e1002987.
